# Estranged relations: coercion and care in narratives of supported decision-making in mental healthcare

**DOI:** 10.1136/medhum-2018-011521

**Published:** 2019-07-30

**Authors:** Meredith Stone, Renata Kokanovic, Felicity Callard, Alex F Broom

**Affiliations:** 1 Social and Global Studies Centre, School of Global, Urban and Social Studies, RMIT University, Melbourne, Victoria, Australia; 2 Royal Hospital for Women, Sydney, NSW, Australia; 3 Hunter New England Local Health District, Tamworth, NSW, Australia; 4 Adjunct, Monash Centre for Health Research and Implementation (MCHRI), Monash Public Health and Preventative Medicine, Monash University, Melbourne, Victoria, Australia; 5 Department of Psychosocial Studies, Birkbeck, University of London, London, UK; 6 Centre for Social Research in Health, UNSW Arts and Social Sciences, University of New South Wales, Sydney, New South Wales, Australia

**Keywords:** psychiatry, sociology, medical anthropology, medical humanities, patient narratives

## Abstract

Supported decision-making has become popular among policymakers and mental health advocates as a means of reducing coercion in mental healthcare. Nevertheless, users of psychiatric services often seem equivocal about the value of supported decision-making initiatives. In this paper we explore why such initiatives might be rejected or ignored by the would-be beneficiaries, and we reflect on broader implications for care and coercion. We take a critical medical humanities approach, particularly through the lens of entanglement. We analyse the narratives of 29 people diagnosed with mental illness, and 29 self-identified carers speaking of their experiences of an Australian mental healthcare system and of their views of supported decision-making. As a scaffolding for our critique we consider two supported decision-making instruments in the 2014 Victorian Mental Health Act: the advance statement and the nominated person. These instruments presuppose that patients and carers endorse a particular set of relationships between the agentic self and illness, as well as between patient, carer and the healthcare system. Our participant narratives instead conveyed ‘entangled’ relations, which we explore in three sections. In the first we show how ideas about fault and illness often coexisted, which corresponded with shifting views on the need for more versus *less* agency for patients. In the second section, we illustrate how family carers struggled to embody the supported decision-making ideal of the independent yet altruistic nominated person, and in the final section we suggest that both care and coercion were narrated as existing across informal/formal care divisions. We conclude by reflecting on how these dynamic relations complicate supported decision-making projects, and prompt a rethink of how care and coercion unfold in contemporary mental healthcare.

## Introduction

‘Care’ is a shifting, plural word when used in the context of discussions of health. It suggests attention and compassion when articulated as a verb, but has overtures of regulation and control when used as a noun.1

Attending to coercion in mental healthcare has arguably become something of a mainstream pursuit. Whereas once it was conspicuously aligned with the antipsychiatry movement, it now publicly occupies an increasingly diverse array of academics, policymakers, clinicians and users of psychiatric services across the Organisation for Economic Co-operation and Development contexts. Much of the recent attention has coalesced around the introduction of the United Nations Convention on the Rights of Persons with Disabilities in 2006, and the supported decision-making framework it endorses.2 Supported decision-making, often presented in contrast to *substitute* decision-making, is a framework built on the premise that all citizens should have the same right to make choices, including about healthcare, regardless of ‘disabilities.’ Furthermore, it emphasises the obligation of mental health systems to provide support so that those who might otherwise have difficulty in, or refrain from, healthcare decision-making can enact their rights.3 This would seem to be an emancipatory project—transforming people previously viewed as mentally disabled into choosing, agentic subjects, and thus by extension reducing coercion in psychiatric care.4 Nevertheless, recent literature is equivocal about the extent to which such a reduction has occurred.5 Notwithstanding the argument that supported decision-making has only of late been adopted on a broader scale, and so its successes are yet to come (and be documented),6 several authors have highlighted difficulties with the implementation of supported decision-making informed mental healthcare. Critiques include that governments have failed to pay sufficient attention to the ‘positive rights’ aspect of the Convention on the Rights of Persons with Disabilities (ie, the right to support, as opposed to the right to not be denied of liberties7); that substitute decision-making remains prevalent even in systems that purport otherwise8; and that people diagnosed with mental illness often fail to use available supported decision-making tools.9

It is this seeming ambivalence of the would-be beneficiaries of supported decision-making projects that we explore further in this paper. To do so we use a critical medical humanities perspective, in particular, the idea of entanglement—the notion that *‘* *both* *medicine and life itself are constituted precisely through relations, and through practices of bordering, cutting and exchange through which those relations come to matter’*.10 This follows on from a number of authors who have recently written on entanglement in this journal.11 Building on this work, and that of other key scholars, we hold that patients and carers are not neutral boundaried actors making linear or predictable healthcare decisions. Rather they exist in, and are formed by, a set of intimate and often contradictory relations with themselves, each other, the outside world, the past and the anticipated future. This may include experiences with healthcare services, historic models of illness and blame, family histories, and expectations of recovery. Decisions are made in dialogue with these relations and others, which as we will see can lead patients and carers on unexpected trajectories. For example, a mother might at once identify as her son’s carer and advocate, and simultaneously maintain that she must make decisions on his behalf because mental illness has rendered him ‘incapable’.

In this example we see how care might easily assume *‘* *overtures* *of regulation and control’* 12 and how the carer becomes an equivocal figure who cares, but does so by restricting freedoms. Such fluid, seemingly incompatible, roles have recently been explored by a series of critical ethnographers and social scientists offering novel perspectives on relationships and ambivalences in healthcare systems.13 Annemarie Mol, for example, has written on how doctors and patients ‘tinker’ together, making decisions informed by entangled histories, social circumstances and illness experiences, often working outside accepted clinical protocols to ‘negotiate health’. Or Hennion and Vidal-Naquet14 have suggested that *‘* *constraint’* (arguably another word for coercion) might sometimes be incorporated into *good care*. In our paper we apply such perspectives in exploring attitudes towards, and enactments of, supported decision-making projects.

### Supported decision-making in the Victorian Mental Health Act 2014

In Australia, the supported decision-making model has been adopted in diverse settings. In the state of Victoria it became a guiding framework for the Mental Health Act introduced in 2014, whose explicit aim was to provide *‘* *assessment* *and treatment (…) in the least intrusive and restrictive way’*.15 For the purposes of the analysis in this paper we have chosen to focus on two key supported decision-making initiatives that appeared with the 2014 Victorian Mental Health Act: the *advance statement* and the *nominated person*. We use these as a scaffolding for our critique:

Advance statements provide information on preferred treatment in the event that a person cannot express their preferences.

A nominated person assists the patient to exercise their rights and can help represent the patient’s views and preferences.

We suggest that these short statements are embedded in a wider set of emergent discourses, logics of care and relations with the modern State.16 The *advance statement* articulates a person with a stable set of preferences, whose capacity for expressing these preferences becomes compromised during an ‘event’. This suggests that expressions occurring *within* the event are non-preferences. In other words, the agentic, choosing subject is intermittently interrupted by a ‘subjectless’ gap.

The role of the nominated person appears to be twofold. One is to *‘* *represent* *the patient’s views and preferences’,* that is the *authentic self*, during episodes. The other role is as advocate, assisting *‘* *the* *patient to exercise their rights’*. The support person appears as an odd hybrid here—at once a relational self with special insight into the patient’s authentic wishes, and a self removed enough to take over when the patient ‘goes down’. Finally, implicit here is a system that *requires an advocate*, a system that, without the nominated person, would tend towards coercion.

We contend that for supported decision-making initiatives to be meaningfully deployed, the intended recipients would need to buy in to, and themselves enact, this ‘supported decision-making version’ of the world. In this paper we suggest that divisions between patient–carer, illness–health, legitimate–illegitimate preferences and system–individuals that are implicit in such a version are only ever partially endorsed by patients and carers. Furthermore, we argue that this ambivalence is crucial to understanding the mixed fortunes of ‘anti-coercive’ supported decision-making practices. In the process, the paper raises unsettling possibilities—for example that patients and carers sometimes desire *pro*coercive practices, or that carers may want to *leave* rather than take over during episodes of illness, or that, intermittently, patients ask for *less* agency. However we simultaneously seek to explain how, from a vantage point of entanglement, such disquieting possibilities might begin to make sense.

In the first section we explore how participants narrated illness and subjecthood, and draw out unexpected conflations of agency, illness and deviance. Here our analysis adds to recent work on the persistence of fault and blame in seemingly neutral models of mental illness,17 in our conjecture that blame provides an implicit justification for coercion or withdrawal of informal care.

In the second we turn our attention to the frequently valorised carer–cared-for relationship. We apply the idea of estrangement (*‘* *The* *splitting away from other bodies, or the internal splitting of a putatively singular body – the making strange of both self and other’* 18) to understand how carer and cared-for can at once be ‘tangled up’, and alienated or in conflict. In doing so we offer a disruptive critique of notions of the objective yet altruistic carer implicit in supported decision-making projects.

In the final section we examine the blurring of formal and informal care practices. We challenge the commonly held idea that coercion is confined to formal psychiatric systems, paying attention both to self-coercion and carer-enacted coercion, but also to how care is sought in the unlikely setting of a psychiatric ward.

## THE STUDY

The findings in this paper are drawn from a large interdisciplinary research project exploring experiences of people in the mental health system, including in relation to supported decision-making. In this paper, we focus on the narratives of 29 people diagnosed with mental illness and of 29 non-linked people supporting diagnosed family members. All had encountered the mental healthcare system in Victoria, Australia. Participants were recruited via advertising through mental health community support services, newsletters and online advertising. Participants’ diagnoses were self-reported (and were most frequently bipolar disorder and schizophrenia). Many had experienced involuntary treatment. All participants provided informed, written consent to be involved in the interview and self-identified as being able to fully participate.

The second author led the study’s interdisciplinary research team, and the fourth author was a member of the project advisory group. The first author is a psychiatrist who has previously worked in the Victorian healthcare system. Interviews were conducted by non-clinical qualitative researchers employed on the study under the supervision of the second author. In the first section participants were asked to provide an account of their experiences of being diagnosed and living with a mental illness, or of supporting a diagnosed family member. In the second section participants were prompted to speak about a range of research-related themes. Themes included diagnosis, hospitalisation, involuntary and voluntary treatments, experiences of making decisions about treatment, or supporting others to make decisions and ideas about recovery. The combination of an unstructured ‘your story’ component and a clarifying section allowed us to identify congruities and divergences in how concepts such as self, agency and illness appeared in different contexts.19

Interviews lasted 1–2 hours and were conducted at locations convenient for the participants. They were video-recorded or audio-recorded with written informed consent, transcribed verbatim, and returned to the participants for review. Importantly, participants could opt to delete sections, which meant that the materials analysed were participant-approved transcripts. Transcripts were subsequently de-identified.

Of note, of the 29 patient participants only 2 people disclosed having made an advance statement and only 2 said they had a nominated person. In the carer arm of the study, one person believed their cared-for was thinking about making an advance statement, and one said that the cared-for had a nominated person.

### Patient and public involvement

The study was supported by an advisory group which included people who had received a psychiatric diagnosis and family carers. The advisory group met on a regular basis for the duration of the study and provided input into the overall project from its design to data analysis. At the end of the study, the advisory group contributed to the translation of research findings into two digital resources aimed at providing information and support to people who have received a psychiatric diagnosis, family carers, educators, policymakers, and health and social care providers.

### Analysis

Our study falls under the umbrella of narrative enquiry, but is indebted to both narrative and thematic analysis techniques. We took *‘* *a* *dialogic approach that advocates an address to content, alongside structure and context’* 20 in order to draw out narrative nuances, while simultaneously capturing a sense of themes occurring across multiple transcripts. To highlight this approach, we present our findings as a combination of collated themes and exemplary vignettes. We also revisit excerpts from a small number of participants throughout the paper to show how seemingly incompatible plotlines (eg, illness histories and stories of deviance) and characterisations (eg, sick vs irresponsible protagonists) became entangled. Of note this is but one of a series of papers we hope to publish from this study—it should function as an introduction to the complexity of themes relating to care and coercion. Elsewhere we will present a more detailed narrative study of selected accounts.

Transcripts were analysed in a series of iterative steps. Initially they were read closely by the first two authors, discussed broadly and emergent themes noted. Subsequently these two authors conducted a genre-traversing literature review, returned to the transcripts, and watched corresponding videos or listened to audio recordings. Finally overarching themes were generated and illustrative quotes selected to create a first draft of the paper. The third and fourth authors joined the writing process, contributing to structure, narrative analysis and the identification of relevant literature.

In interpreting the narrative data we combined an interest in entanglement21 and ‘hauntological relations’ (after Karan Barad22—the idea that what is ‘now’ and ‘here’ are continually disrupted by what is/was ‘there’ and ‘then’) with related attention to how discourses shaped (haunted) participant narratives.23 Although our use of video and audio material provided the backdrop to some of the analysis, and hints of the affective reverberations in the research process, we have not included them as ‘data’ in this paper. This, combined with the fact that the authors did not conduct the interviews themselves, means that we offer few reflections on *entangled research processes*. Thus we do not seriously broach co-construction of narratives between researcher and interviewee, affective/discursive/material entanglements, or reflections on how our own professional histories transform/are transformed by encounters with ‘data’. Again, we intend to address these aspects elsewhere.

### Theoretical considerations and a note on terminology

We reflected at length on how to describe our participants. Ultimately, we decided to use ‘neutral’ terms such as participant where appropriate, but to apply terms such as carer, cared-for and patient in circumstances where this was the dominant narrative positioning we could discern (eg, where participants described themselves or the protagonists in their narratives as carers/patients). Nevertheless, a key intention is to simultaneously reveal how such terms structure experience and how experience destabilises these terms (eg, the carer who is absent during illness episodes).

Our use of care and coercion is deliberately ambiguous. Care we conceive of interchangeably as the acts of a designated carer, as being in relation24 *and* as being concerned about or preoccupied by something.25 Coercion we consider by degrees, drawing on recent theorising about how diverse attempts to influence an other (or oneself) can be conceptualised on the ‘coercion spectrum’. This is a spectrum that encompasses ‘persuasion’26 and ‘nudging’27 alongside more widely recognised forms of psychiatric coercion. We do not offer a new dogmatically plural understanding of these concepts or seek to relativise the excesses of restraint and control on psychiatric wards. Rather we hope to engender novel ways of ‘thinking through’ what counts as care and coercion, and to ask how such reclassification would complicate seemingly neutral or unambiguous relations.

## Suspended selves or willed illness?

We begin our analysis by exploring how participant narratives corroborated or contradicted the self–illness split underpinning the advance statement. We have devoted a section to this for two reasons. First, the anticoercive function of the advance statement is of course precarious. It operates not by avoiding all coercion, but by accepting that upholding a ‘well’ person’s preferences will likely entail going against their wishes during ‘episodes’. It is only if we accept that the wishes during such episodes are spurious, *mere symptoms*, that the advance statement can indeed be anticoercive.28 Furthermore, in Australia, as elsewhere, emphasising the split between illness and person is often used to counter beliefs about deviance.29 Moreover, the split is invoked to advocate for *care as opposed to coercion* for those diagnosed with mental illness, by asserting that mental illness is like any other and *is not the diagnosed person’s fault*.30 This of course compels the question—how would these arguments change if the person were seen not to be separate from their illness, to have some control over what happens during ‘episodes’? And how might this unsettle the conversation about choice, freedom, care and coercion?

### Not at fault for being sick but responsible for staying well

In our narratives the self–illness split was superficially endorsed by many participants. For example, Brittany emphasised that those diagnosed with mental illness *have “something wrong”* as opposed to *are “crazy.”*

People with experience of [mental illness] think well why is this happening to me? (…) They feel like they’re going crazy where you’re not going crazy. You actually have got something wrong with you. (Brittany)

Alejandra also clearly distinguished between personhood and illness:

It’s very hard to realise that you’ve actually had episodes of being unwell where you’ve done things and behaved in certain ways and smelt things and heard things which are all not real (…) But I think once you come to terms with it and try and realise that it’s not your fault, that it is your illness you know you do feel quite a bit better about it. (Alejandra)

However in repeated readings of the transcripts we noticed that the explicit narrative position, often emphatically conveyed as can be seen above, could conceal moments of ‘narrative doubt’ or hidden meanings. Models of illness and selfhood, agency and fault were thus more complicated than they initially seemed. The narrative blurring of self and illness occurred in many ways. One subtle contradiction was that while many participants, like Alejandra, emphasised that people could not be blamed for their illness, the participants simultaneously felt it was their responsibility to ‘stay well’:

I guess I’m lucky that the medication works and I can have normal thoughts and I don’t have any episodes.

But that’s not saying I might not in the future (…) I have to monitor my stress level to make sure that, you know, I adjust my life accordingly and don’t get into situations where, you know, I feel like things are out of control again. (Alejandra)

This extract begins in the tenor of Alejandra’s previous one, referencing ‘luck’, and thus implying that illness and self are independent entities, randomly connected. However, after a pause Alejandra introduces the idea that she *does* have some responsibility for her illness, paradoxically intimating that were she to relapse, she could not be deemed to be entirely faultless.

### On preferring to remain ill: accounts of agency during illness episodes

In our narratives ‘ill’ protagonists were not, as we had expected, always depicted as hapless figures afflicted by a narratively demarcated illness. Rather symptoms often became absorbed into the character of the protagonist and temporal delineations between ‘healthy’ and ‘ill’ difficult to discern. This process can be seen in a section of Wendy’s (carer) account, where she described how her son had deceived his doctor during an illness episode and thus avoided necessary hospitalisation:

Wendy: S might have been putting on a good show to the doctor, which they can do. They can act quite normal and responsible and convince you of what they can do and can’t do and it’s not the truth.

Interviewer: Do you think at that time, they are making an effort to behave in that way? Or are they actually just well?

Wendy: Oh they do, no, no they – they know what goes on, they learn from the others, what goes on, how you should behave. And if you get the right people there, that you can pull the wool over their eyes, you’re right. So he did, he was lucky that day, or unlucky for us.

This vignette shows what we might call an entangled, or ‘mixed-up’, illness model. Wendy clearly in part endorses a biomedical frame for understanding S’s problem; she asserts that S is not *“well”* and that his rightful place during an episode is in hospital. Nevertheless, she also imputes qualities of an agentic subject—evoking a character who despite being ill can plan and organise and *wilfully deceive*. Furthermore, she disrupts the temporal and spatial delineations characteristic of a biomedical model. When she discusses how *“they know what goes on, they learn from the others,”* we get the impression that S has planned his behaviour and has associated with ‘other patients’ *before* the ‘episode’ and when not in hospital. Ultimately, we are left uncertain whether he is *ever* well, or in fact, given that his capacity to learn from ‘them’ and implement ‘their’ techniques seems uninterrupted, whether he is *ever unwell*.

### At fault after all

This narrative ambiguity around illness episodes versus (implicitly deviant) agency was surprisingly common. Several participants, in both arms of the study, spoke of how cared-fors or they themselves had lied, performed or deceived during ‘episodes’. Furthermore, as can be seen with Wendy, although most participants ‘officially’ endorsed a biomedical temporality of healthy self—illness episode—healthy self, many narratives failed to clearly punctuate these transitions. A notable corollary was uncertainty around whether decisions made when ‘unwell’ were irrational or *irresponsible*:

You make decisions and you take the responsibility too, but this mental illness, they make irrational decisions. They do not take responsibility (…) and they get away with it (…). (Hannah, carer)

Hannah’s emphasis here on *“getting away with it”* suggests that those with “*mental illness”* are capable of acting otherwise, indeed that they *choose* their actions according to anticipated benefits. Penny, a carer of an adult son with a diagnosis of psychosis, was similarly preoccupied with how her son’s irresponsibility had led to his current episode of illness and to hospitalisation:

We try to focus on the fact that we’ve got to care for ourselves and, and J now has to take more responsibility. We’re not there to pick up the pieces all the time, he’s not a little boy anymore, he’s an adult. (Penny, carer)

These narratives provide a somewhat foreboding response to Alejandra’s concern at the beginning of this section that if she did not look after herself, she could be held responsible. They seem to suggest that you can and *will* be held to account for not doing more to prevent an episode. The denouement to Hannah’s care narrative, in which she described why she no longer felt guilty about not being there in the period preceding the suicide of her stepdaughter, T, perhaps illustrates this most starkly. In one section of the narrative Hannah conveys that T was ill before she died:

Actually the last time when I saw her, when she went crazy last year, she was at the front door, she was carrying on, she was crazy (…).

But then elsewhere Hannah emphasises:

I will not take it on board sort of feeling guilty about it, or sad about it anymore. It was her choice at the end of the day, I feel, you and only you can make decisions in your life and choices. So it is your responsibility. (Hannah, carer)

These narratives reveal some of the difficulties with the supported decision-making model of a subject with ‘legitimate’ preferences interrupted by illness. The participants in our study clearly employed polyvalent, fluid models to understand how ‘episodes’ emerged. Illegitimate preferences were therefore not just seen as manifestations of an illness episode, as suggested by the supported decision-making model, but for many seemed to have *caused the episode*.31

Our paper thus adds to the literature on how medical models can coexist with, indeed co-constitute, seemingly anachronous models of blame.32 Significantly, our findings build on this work by emphasising that it is not just that those diagnosed with mental illness continue to blame themselves, or that family members continue to experience blame, but that blame *moves between* people, and that, sometimes at least, carers blame the cared-for. In terms of entanglement they reveal the uncertainty our participants expressed about what was ‘normal’ and what was ill—the mingling of ‘behaviours’ and ‘symptoms’, choice and arbitrary hand of fate. But also to entanglements in the hauntology sense of the word—the idea that beliefs about deviance do not just ‘live in the past’ but ‘flash up’ in the present.33 That they haunt because they are not allowed to be said,34 must be couched in the available blameless language. Thus, Brittany and Alejandra can emphatically declare that *“it’s not your fault”* or *“you’re not going crazy”* and in doing so ‘flash up’ the possibility that it *could have been* your fault or that you *might have been* going crazy. And Hannah can appear to be lauding choice and agency, and simultaneously be telling the unsettling story of how her *cared-for* had been alone in the period before she ended her life.

Finally then, this section has itself begun to say something that *ought not to be said*—that in the carer–cared-for relationship blame, alienation and ‘stepping back’ can exist alongside, or even supersede, altruism, advocacy and ‘taking over’. We now address these themes more concertedly, tracking how the carers and cared-fors in our narratives were both entangled *and* estranged.

## Whose preferences?

But the wider issue for all of us is how to rethink the modernist assumption that the embodied subject is autonomous and distinct from her others, and contained by the boundaries of her own body.35

There have been very few people in A’s life that have actually listened to me and supported me. (Erin, carer)

### Illness in relational context

In our introduction we suggested that the supported decision-making model presupposes the presence, and involvement, of an empathic, ‘in-tune’, but ultimately boundaried and ‘sensible’ carer. In our interviews, some carers did offer narratives consistent with an advocacy and ‘back-up self’ model of support.

However, in other narratives the premises of the supported decision-making model, and much of the broader literature on carer involvement in mental healthcare, were destabilised. In the literature there is an increasing focus on the difficulties faced by informal carers,36 but such difficulties tend to be perceived as resulting from the destructive force of a biomedical illness arriving in an otherwise intact family. For example:

Illness creates a need for increased competence in coping with problems or maneuvering in health services and adds challenges in maintaining interconnectedness and relationships among family members.37

There are of course compelling historic reasons to focus on aberrant neurotransmitter pathways and genes, as opposed to aberrant families, when considering why it is troublesome to care for someone with a psychiatric diagnosis. But as Callard *et al* 38 in their discussion of schizophrenia note, the *‘* *fear* *of giving any energy to discredited models of family blaming’* can limit *‘* *the* *kinds of conversations it is possible to have about Schizophrenia.’* In our study the conversations participants engaged in about schizophrenia (or other mental illnesses) were complex. Importantly, just as descriptions of illness episodes were not ‘emptied of subjects’, they were not relationally neutral.

Penny, for example, whose narrative we introduced in the first section, in this excerpt justifies why she needed to *“step back”* from caring:

It has made me feel a little bit more comfortable in the terms that we’re taking a step back and giving it, the responsibility, to him. He’s the one that has to do the hard work and we’re just hoping that it – see, nothing else worked, all the love and the support and the, you know, and being there and taking, you know, frozen dinners to him [laughs] (…). (Penny, carer)

In this excerpt Penny expands on why she believes her son should ‘take more responsibility’. Notably there is a profound sense of disappointment that Penny’s care was *not enough,* that she was doing the *“hard work”* and this work was not reciprocated. J’s illness therefore is not a neutral collection of symptoms, but rather, in its very existence, speaks to Penny about *who she is* and what she as mother and carer has or has not achieved.

### Caring in an outcome-oriented world

This vignette shows how accounts of illness became tangled up with stories of relationships. However, it also destabilises the idea of ‘pure’ carer–cared-for relations. Penny provides a compelling account of what it feels like to be a maternal carer whose son *refuses to get well*. Her narrative disrupts ideas implicit in the supported decision-making imaginary39 of the carer as altruistic, self-displacing, relationship-suspending advocate, a figure quarantined from the vagaries of an outcome-oriented world. Instead Penny’s account points to how that outcome-oriented world can interrupt, estrange, co-constitute the carer–cared-for relationship. This manifests in different ways. Several carers (usually parents) mentioned how promising the lives of their cared-fors had seemed ‘before illness’. But participant narratives differed in how they narratively resolved, or, importantly, *failed to resolve*, such ‘lost futures’.40 In accounts such as Penny’s and Hannah’s, there was a sense that the cared-for had not fulfilled their part of the ‘care contract’. Both participants emphasised how much they had done for their children and how bright their future *could have been*:

I’d say the world wasn’t big enough for her, for the plans that I had for her (…). (Hannah, carer)

And both seemed to, implicitly at least, blame the cared-for, not just for ‘becoming ill’, but for doing so despite—*in spite of*—their caring attentions. Penny reinforced this idea by describing the ‘successful’ trajectories of her two other children, whose repeated reassurances that *“it’s not us that have caused (J’s illness),”* and entreaties to stop ‘caring so much’ for J, provided a narrative backdrop to J’s episodes. Far from supporting popular notions of carer–cared-for attunement, these narratives seemed to present an *‘* *imperative* *estrangement (…) powerfully articulated as the denial of* *loss* *’* 41 (of the hoped-for, self-extending successful child).

### Carers versus cared-fors?

Other narratives also hinted at the permeability of boundaries between the carer–cared-for dyad and the ‘outside world’. Surprisingly, several carer participants framed their personal struggles with references to an inherent, seemingly inevitable, conflict between carers and ‘the mentally ill’:

[mental health care systems] don’t take the side of the victim. They take the side of the perpetrator (…) The person who’s mentally ill is the perpetrator. (Amelia, carer)

For these participants, the carer–cared-for dyad was located within a broader system of constitutive (and conflictual) relations, a theme that is also beginning to be explored elsewhere in the literature on informal care.42

Remarkably though the narratives in our study were *not* of radical separation. They were narrated by participants *who continued to identify as carers*. This paradox is illustrated well by Amelia, when she continues:

And where does the law go? With the perpetrator. That’s how it feels at times, you know. And your only intent is to help, help the person you love.

Thus Amelia, Penny and Hannah describe estrangement but from *a starting point of entanglement*. In their narratives we see attempts to distance themselves, to not feel guilty, to defend against ‘a perpetrator’, but these are the narrative allies of love (Amelia), adoration (Hannah) and delivering frozen dinners (Penny). We get the sense that such manoeuvres are an endeavour to ‘border, cut and exchange’43 in a relationship where the beginning and end of carer and cared-for are hard to distinguish. Narratively we see this in the merging of carer and cared-for fortunes: for example in Penny and Hannah’s preoccupation with the failure of their caring endeavours to prevent the cared-for’s illness, or in Erin’s blurring of protagonists in the quote opening this section (*‘* *There* *have been very few people in* A’s life *that have actually listened* to me *and* supported me”).

### Too close to care?

Interestingly even among the participants who narrated a more expected narrative configuration of the altruistic carer and care-requiring cared-for, there were some who felt that the carers were ‘too close’ to act as effective advocates in mental healthcare systems:

And some of that is my fault, I suppose. Should I have pushed, should I have dragged him off to places, should I - but you couldn’t do that, he just absolutely refused (…) I was afraid that I would lose him altogether. (Larissa, carer)

Not only because he’s a professional but because he knows me and I don’t feel like, you know, for example my mum would be able to separate that emotional side. (Donna)

I am unwilling – even though I know he should have that information – I am unwilling to have that discussion with the doctor, because if he tells my son that I’ve told him that, my son won’t allow me in. (Natalia, carer)

In such narratives the contradictory expectations on informal carers within the supported decision-making logic became apparent. Implicit in supported decision-making projects seems to be the idea that carers will act as reservoirs of intimacy and authentic knowledge, but also that these reservoirs will function as *resources* for the formal psychiatric system.44 This is exemplified in an ancillary Victorian government website detailing the role and importance of mental health carers: *‘* *families* *and carers often have knowledge that is essential information for clinicians in their assessment’.* 45

It is assumed that informal carers will ‘report back’; identify warning signs and ‘relapse signatures’; extend the gaze of clinicians. Furthermore, in their admission that they are too emotional, worried about losing their sons or not being ‘allowed in’, these participants, paradoxically, seem to be conceding a caring failure: *“* *even* *though I know he should have that information”*; *“and some of that is my fault I suppose.”* This reminds us of Shildrick and Steinberg’s observation that *‘* *estrangement* *can be both the effect of, and resistance to, governance’*.46 Larissa and Nathalia perceive themselves (or are perceived by others) as deficient (*strange*) carers because their caring activities, at least at times, occur in opposition to formal psychiatric systems. As Donna’s excerpt suggests, they fail as capital *c* Carers because they *care too much*. Thus as they attempt to ‘return’ to some form of authentic or intimate caring, they narrate themselves as deviant.

## The system is everywhere, but not where I need it

Such analysis suggests that the ‘uncaring’ excerpts we have used in this paper *may not be as aberrant as they seem*. What if there is an element of uncaring, of distance, of conflict *built into* the figure of the informal carer? In this final section we explore the idea of the informal carer who, *in caring*, enacts coercive functions. However, in keeping with the idea of entanglements, of porous boundaries between system, carer and cared-for, we go on to suggest that ‘familial style’ care is conversely sometimes sought in formal psychiatric services.

### Informal carers and coercion in formalised care

In several of our narratives there were descriptions of psychiatric wards in which patients and carers felt disempowered and unable to influence the course of treatment. A graphic example was Geraldine’s account of her experiences in seclusion:

I was in seclusion and I was in there for days. And in between that I, there were parts where I was brought out and strapped to a bed. But I was really - they had to use security a few times to hold me down to put – to give me a needle and I just had bruises all over me. (Geraldine)

That the mental healthcare system depicted here tends towards coercion and disempowerment is evident. However, Geraldine’s narrative was also notable for the role informal carers had in relation to the system. Geraldine felt that her ‘support people’ were instrumental in getting her admitted to hospital and in keeping her on the ward for a longer period:

I was never allowed to know what they had been saying to the doctors either and they continued to report on me. So eventually I was allowed on days out in [city] and I only had two girlfriends to go out with and they were taking me out and then ringing and reporting to the doctor.

Of note here is the way in which informal (and apparently self-anointed) carers and the hospital staff are seen to interact, in fact almost conspire, to remove the rights of the person who has been diagnosed with mental illness.

### Coercion at home

In other narratives coercion appeared in concealed ways. A noteworthy example was in Ben’s account. Ben described an all-consuming caring role for his wife and advocacy against a disempowering mental health system. He gave a lengthy account of how his wife’s confusion on a locked psychiatric ward had been misdiagnosed as psychosis, when he, as long-time husband and carer, knew that it must be related to her periodic bouts of constipation. He detailed how after days of presenting this information but not being heard, and his wife’s condition worsening, he smuggled in laxatives and the problem was solved in a dramatic and, for the hitherto disbelieving nurses, rather messy way. Ben followed up this anecdote by asserting that his wife would never again be admitted to a psychiatric ward. In the dominant narrative arc, then, there was a portrayal of a self-sacrificing, attuned carer, with concerted and apparently faultless advocacy against a powerful system. Yet what became apparent through the course of the interview was that his solution to a locked psychiatric ward was not an eschewal of the healthcare system, but a transplantation of that system into the home:

I printed off from the internet a bowel movement chart and copied it and laminated it and put it in with a felt pen in the toilet, so that she can make a mark every time that she’d actually had a bowel motion.

When they want to change the dosage, even by a few milligrams, it’s like, I don’t know, just ask him (Ben). Because I have to keep a total track on what’s going on and when they’re going to play around and it’s like I’m half doctor, half psychiatrist and another half of me – yeah, that’s three halves – is a pharmacist.

And although this project was described as an altruistic endeavour, sometimes there were unsettling aspects to the narrative. Ben’s response, for example, when asked whether his wife had an advance statement was:

My wife’s advanced statement is me, “ask him” [laughs]. Yeah, it’s simple as that, because unless I tick the box, it ain’t going to happen.

And in his description of daily life:

And you can twist it too. I love it when I say, “Hey, don’t forget, you just said you were going to go and make your coffee” and she hadn’t at all, but, “Oh, did I? Sorry.” Up she gets, goes and makes a coffee. I can do it all day long, I don’t have to get up and make one coffee.

In these excerpts Ben’s wife is not physically located in a psychiatric ward, but we wonder whether she is free of the mental health system, or indeed of coercion. As we have reflected before,47 care here ‘takes on an odd face indeed’, a face, moreover, that remains largely obscured in the literature on mental health and informal care.

This vignette powerfully illustrates how the system and its coercive practices are not confined to formal psychiatric wards. Indeed traces of coercion were found in divergent narrative settings and on unlikely protagonists. For example, in the patient arm of the study there were a number of ‘consumer consultants’ or ‘peer workers’ who volunteered to be interviewed. In these interviews, the narrative voice frequently shifted between that of ‘lived experience narrator’ (someone with their own experience of mental illness) and that of ‘expert informant’ (someone narrating as a mental health system employee), the latter position often appearing to be more comfortable. In expert mode, advice, or even admonitions, became an integral part of the narrative performance. This is demonstrated well in this section of text where Alexis promotes the benefits of psychotropic medication:

People think once they’re on medication and they get better (…) they think that they’re better automatically. They think they should stop their medication. But they don’t realise it’s the medication’s made them better. (…) That medication, if it makes him well, it should be respected.

Alexis’ narrative reveals slippages between the categories of patient and professional, and shows how ‘soft forms of coercion’48 could be enacted by people diagnosed with psychiatric disorders, both on themselves and others.

### Familial care in the psychiatric clinic

Interestingly, Alexis elsewhere described a close relationship to his doctor:

The one that I’m having now I’ve had for maybe 20 years or more. He’s a friend of mine, a good friend of mine now, rather than a practitioner (…) Calls me his best patient, isn’t that cute?

The depiction of psychiatrist or case worker as friend or paternal/maternal figure was not unusual. Furthermore, several participants expressed the view that they could be ‘more open’ with clinicians or support workers than with family when making decisions about treatment.

These narratives thus challenged conceptualisations of the family as inherently in-tune and better at knowing what a cared-for might want. They resonate with other literature documenting how relationships with professionals can sometimes take on familial forms and become a reservoir for feelings and modes of relating that are unable to be accommodated in the nuclear family.49 In our study this relating to clinicians as though they were family was extended further. Many seemed to feel that patients ultimately belonged in hospitals:

I feel that carers won’t be hanging around to care if they’re unable to liaise in a productive manner with the medical staff, because they’ll be throwing their hands up in the air going okay, well you want to deal with it, you don’t want me to be involved, you have it all then. I’m trying to help you in your job and have this person at home and not in the hospital system. (Rachel, carer)

“They are probably going to send him home, Friday.” I said, “Well, in that case, you better tell him, ‘Don’t come home, because I will change the locks.’ (…) Tell the psychiatrist, ‘Take him home to his house with a new baby and see how he copes.’” (Amelia, carer)

(On being asked about the changes to the Mental Health Act) That is just a buck-passing invention to pass some of the responsibility or more of the responsibility off the mental health system, onto the carer. (Micheala, carer)

These excerpts show that for many carers ‘the system’ was not perceived as a service provider to be called on in times of crisis or in an advisory capacity. Rather the system was seen as having shared or even *primary* responsibility for those diagnosed with mental illness, and carers were constructed as the ones ‘helping out’. Here then a resistance to the ‘responsibilisation’ of informal carers50 becomes apparent as biomedical narratives are used to ‘return’ patients to their ‘proper place’. Entanglements become not just about the flow of the exterior into the interior, the psychiatric system into the intimate, but also about its obverse—the movement of familial responsibility into psychiatric services, the demand that the psychiatrist take the patient home, that mental health systems stop *“* *buck* *-passing”* onto carers.

Furthermore, significantly, it is not clear in these narratives whether the carers are requesting more care or more coercion (ie, longer periods in involuntary treatment) *or both*. In part this speaks to the already addressed theme of conflict between carers and cared-fors, to the disruptive possibility that to *care for carers*, there ‘needs’ to be *more coercion* in mental health systems. However, it also returns us to our starting premise that *‘* *care* *is a shifting, plural word’* 51 and to the even more disruptive possibility that care is *necessarily* entangled with coercion. Importantly this idea was not just conveyed in the carer arm of the study, but was also implied by several patient participants. Often the absence of ‘minor coercive tactics’ was construed as something of an abandonment or lack of care. This was illustrated by Dorothy when discussing how her children should have been more assertive in ‘getting her help’:

They kept on saying, Mum, the only person that can help you is you. And, yes, in a way that is true, but in another way, no, you need somebody to give you a kick up the pants. (Dorothy)

Or by Justin when he compared two different clinicians and maintained that the “bad one,” who had gone along with everything he said, “didn’t care”:

If you don’t care about them enough to dispute something with them, then you’re not doing your job. (Justin)

Such accounts demand a shift in how we think about the intersection of care and coercion in mental healthcare systems and further trouble the idea of the boundaried, self-knowing subject who generates preferences independently of the relational context.

## Coercion by omission and other conclusions

In this paper we have introduced a framework for understanding the contradictions inherent in contemporary supported decision-making projects. Our analysis has revealed how a supported decision-making version of illness, patient, carer and system at times structured our participant narratives, but that this structure simultaneously obscured more complex, ambiguous and fluid relational dynamics. Notable was the way in which supposedly outdated models of blame slipped into, and were narratively accommodated in, ‘blameless’ narrative trajectories.

That such paradigm slippages have a material impact on supported decision-making enactments was endorsed by our narratives. Thus many of the patient participants were able to describe the potential benefits of advance statements, but admitted to not having one themselves. As Abigail ‘explained’:

This might sound - I don’t - I - I’m - yeah, I haven’t - I don’t plan on becoming really unwell again [laughs].

The failure to make advance statements is illogical if we assume that those diagnosed with mental illness are rational actors with intermittent biochemically induced suspensions of reason. But if instead we believe that illness episodes are *moral failings*, indicative of insufficient effort to recover,52 then advance statements present an impossible bind—at once denoting the rational, self-monitoring, hazard-averting citizen, and introducing the possibility that her efforts will not be good enough.

Similarly, if we consider the informal carer not as inherently altruistic, with ‘authentic’ knowledge of the cared-for, but rather as someone who has been *called into being* by psychiatric systems, then the advocacy project becomes a formidable, often unrealisable undertaking. This too was borne out in our study. Whether in Geraldine’s account of the friends who rather than shortening her hospital stay, prolonged it, or in Ben’s description of *substitute* decision-making, or in Larissa, Donna and Natalia’s stories of carers becoming ‘too emotional’ to effectively advocate. Perhaps most startling were the descriptions of carers who simply *were not* *there* when an ‘episode’ was occurring. If we consider the complex relations conveyed in our narratives, the mingling of self-blame and projected blame, *the unbearable closeness* of a cared-for who cannot be cared into wellness, such ‘stepping back’ perhaps represents the embodiment of a ‘broken care narrative’ (a narrative that is seemingly derailed or disrupted53). This idea of unbearable closeness adds to, hopefully productively disrupts, the existing body of literature on carer involvement in supported decision-making. It suggests that if we are to speak of relational selves,54 or ‘intimate’ knowledge and affect flowing between carer and cared-for, then we must also take seriously how the intimate is in constitutive relations with ‘the outside,’ and how blame, guilt and anger *also flow*. Furthermore, these flows are not just thought or felt, but are enacted in ways that, as we have seen, complicate supported decision-making projects.

Surprising too were the participants, in both arms of the study, who, when asked about supported decision-making, responded not with accounts of explicit coercion, but with narratives of abandonment, *lack of care* or even a desire for *more coercion*. This is not to say that coercive practices no longer exist on psychiatric wards, or that supported decision-making projects are redundant. Indeed, our own narratives revealed the persistence of ‘traditional’ forms of coercion. But rather it is to reassert that in rationalised healthcare systems traditional ways of coercing exist as part of a larger, distributed, network of governance, of self-sanctioning, or of carer and peer worker regulation. Moreover, we contend that ‘formal’ coercive practices on psychiatric wards, with their unwieldiness, costliness and their visually perceptible affront to humanist ideals, are not the system’s *preferred form of coercion*. And so for many of our participants their experiences of mental health systems were as much about being told to *go away*, as being deprived of liberties. That this experience of mental healthcare shaped responses to the supported decision-making project was evident. Thus, as we have seen, there was a deep scepticism among some participants, a belief that there were hidden motives behind supported decision-making initiatives, that they were part of a mental health architecture seeking to ‘offload care’. And for others we had the sense that we were *asking the wrong questions*; that supported decision-making was an interesting theoretical exercise; but that having agency in making decisions was not their biggest concern; that what worried them most was *“not that others boss you about, but that nobody cares.”55*

Hence our findings are located within a growing body of work critiquing the ideals of the choosing, self-realising patient and healthcare systems where ‘choice’ (or preferences) is paramount.56 Furthermore they prompt questions we believe have yet to be concertedly addressed. Why has supported decision-making become so popular at a time when the number of involuntary psychiatric beds in Victoria (and elsewhere) is historically low?57 Is it a coincidence that supported decision-making was introduced at the same time as the increased emphasis on carer participation in the Mental Health Act? To what extent *is* supported decision-making part of a minimalist mental health architecture ‘offloading care’? What questions do we *not* ask when discussing agency and preferences and individual rights?

This last question finds echoes in recent work on the complex relations between care and coercion.58 As Hennion and Vidal-Naquet59 point out, *‘* *constraint* *’* to date has generally been seen as a ‘necessary evil’ in discussions of mental healthcare, but what if, more radically, constraint is part of good care? What if, as Justin and Dorothy suggest, *none of us* know exactly what we want? Our preferences change, contradict each other, are co-constituted by (and resist) discourses and embodied others, are enacted in ‘caring assemblages’60? Then perhaps providing *“* *assessment* *and treatment (…) in the least intrusive and restrictive way”* is *not always* consistent with good care.61 It might rather, as we saw in our narratives, be experienced as a *failure to care*. Or even, for participants such as Amelia, with her narrative of perpetrators and victims, as a *violent omission*.

Likewise, we saw traces of resistance in our narratives. For example in the accounts of carers who threatened to *“* *change* *the locks”* or who told mental health services they could *“have it all,”* who were *ir*responsible as *“* *a point* *of intervention*.”62 These are practices whose dynamics are only hinted at within the limits of narrative. We hope that future research might from the outset employ plural methodologies that better capture how ‘care talk’ correlates with ‘care practice’, drawing perhaps on the ethnographic work of Mol,63 Pols,64 and Hennion and Vidal-Naquet,65 or on the thoroughly interdisciplinary and entangled projects filtering into the critical medical humanities.66 For now, we hope this paper will contribute to a broadening of the ambit, a diversification of what counts in examinations of care and coercion.

Finally then, this paper is not a call for supported decision-making projects to be dismantled, or for a return to the ‘good old days’ of paternalism and psychiatric force. Rather it aspires to Latour’s idea of ‘*critique as a form of disruptive rebellion’*,67 to a reimagining of the complex relations of care and coercion in contemporary mental healthcare, and to a seeing, feeling and connecting of the unexpected valences in supposedly empty gaps and omissions.

## Notes

1. Stuart Murray (2016), “Afterword: Health, Care, Citizens,” in *The Edinburgh Companion to the Critical Medical Humanities*, eds. A. Whitehead, A. Woods, S. Atkinson, J. Macnaughton and S. Richards (Edinburgh: Edinburgh University Press), 627.

2. Gavin Davidson et al. (2016), “An International Comparison of Legal Frameworks for Supported and Substitute Decision-Making in Mental Health Services,” *International Journal of Law and Psychiatry* 44: 30–40; Gavin Davidson et al. (2015), “Supported Decision Making: A Review of the International Literature,” *International Journal of Law and Psychiatry* 38: 61–67; Piers Gooding (2013), “Supported Decision-Making: A Rights-Based Disability Concept and Its Implications for Mental Health Law,” *Psychiatry, Psychology and Law* 20, no. 3 (2013): 431–51; George Szmukler et al. (2014), “Mental Health Law and the UN Convention on the Rights of Persons with Disabilities,” *International Journal of Law and Psychiatry* 37: 245–52.

3. Gooding, “Supported Decision-Making”; Fauzia Knight et al. (2018), “Supported Decision-Making: The Expectations Held by People with Experience of Mental Illness,” *Qualitative Health Research* 28, no. 6: 1002–15.

4. George Szmukler (2015), “Compulsion and ‘Coercion’ in Mental Health Care,” *World Psychiatry* 14, no. 3: 259–61.

5. Kirsten Bell (2015), “Thwarting the Diseased Will: Ulysses Contracts, the Self and Addiction,” *Culture, Medicine & Psychiatry* 39, no. 3: 380–98.

6. See Gooding, “Supported Decision-Making.”

7. Gooding, “Supported Decision-Making”; Bernadette McSherry and Kay Wilson, 2015, “The Concept of Capacity in Australian Mental Health Law Reform: Going in the Wrong Direction?” *International Journal of Law and Psychiatry* 40: 60–9.

8. Alan Quirk et al. (2012), “How Pressure Is Applied in Shared Decisions About Antipsychotic Medication: A Conversation Analytic Study of Psychiatric Outpatient Consultations,” *Sociology of Health & Illness* 34, no. 1: 95–113.

9. Bell, “Thwarting the Diseased Will.”

10. Des Fitzgerald and Felicity Callard (2016), “Entangling the Medical Humanities,” in *The Edinburgh Companion to the Critical Medical Humanities*, eds. A. Whitehead, A. Woods, S. Atkinson, J. Macnaughton and S. Richards (Edinburgh: Edinburgh University Press).

11. Julia Kristeva et al. (2018), “Cultural Crossings of Care: An Appeal to the Medical Humanities,” *Medical Humanities* 44, no. 1: 55–8; Brandy Schillace (2018), “Editor’s Note: Pain and Paradoxes,” *Medical Humanities* 44, no. 2 (2018): 71; Margrit Shildrick et al. (2018), “Messy Entanglements: Research Assemblages in Heart Transplantation Discourses and Practices,” *Medical Humanities* 44, no. 1: 46–54; William Viney et al. (2015), “Critical Medical Humanities: Embracing Entanglement, Taking Risks,” *Medical Humanities* 41: 2–7.

12. Murray, “Afterword,” 627.

13. Lucy Burke (2016), “On (Not) Caring: Tracing the Meanings of Care in the Imaginative Literature of the Alzheimers Epidemic,” in *The Edinburgh Companion to the Critical Medical Humanities*, eds. A. Whitehead, A. Woods, S. Atkinson, J. Macnaughton and S. Richards. (Edinburgh: Edinburgh University Press, 2016), 596–610; Antoine Hennion and Pierre A Vidal-Naquet (2017), “Might Constraint Be Compatible with Care? Home Care as a Situational Ethics,” *Sociology of Health & Illness* 39, no. 5: 741–58; Anne-Marie Mol (2008), *The Logic of Care: Health and the Problem of Patient Choice* (London: Routledge); Anne-Marie Mol et al. (2010), *Care in Practice: On Tinkering in Clinics, Homes and Farms* (New York: Columbia University Press)Murray, “Afterword”; Jeannette Pols (2003), “Enforcing Patient Rights or Improving Care? The Interference of Two Modes of Doing Good in Mental Health Care,” *Sociology of Health & Illness* 25, no. 4: 320–47.

14. Hennion and Vidal-Naquet, “Might Constraint Be Compatible with Care?”

15. Vic.gov (2014), “Mental Health Act 2014,” last modified 2018, https://www2.health.vic.gov.au/mental-health/practice-and-service-quality/mental-health-act-2014

16. Brian Brown and Sally Baker (2013), *Responsible Citizens: Individuals, Health and Policy under Neoliberalism* (London: Anthem Press); Virginia Held (2005), *The Ethics of Care: Personal, Political, and Global* (Oxford: Oxford University Press, 2005); Virginia Held (1995), *Justice And Care: Essential Readings In Feminist Ethics* (New York: Westview Press).

17. Bell, “Thwarting the Diseased Will”; Felicity Callard et al. (2012), “Holding Blame at Bay? 'Gene talk' in Family Members' Accounts of Schizophrenia Aetiology,” *Biosocieties* 7, no. 3: 273–93.

18. Margrit Shildrick and Deborah Steinberg (2015), “Estranged Bodies: Shifting Paradigms and the Biomedical Imaginary,” *Body & Society* 21, no. 3: 6; see also Shildrick et al, “Messy Entanglements.”

19. Molly Andrews et al. (2008), “Introduction: What Is Narrative Research?” in *Doing Narrative Research*, eds. M. Andrews, C. Squire and M. Tamboukou (London: Sage), 1–26.

20. Andrews et al, “Introduction: What Is Narrative Research?”

21. See also L.-C Hyden (2018), *Entangled Narratives: Collaborative Storytelling and the Re-Imagining of Dementia. Explorations in Narrative Psychology* (New York: Oxford University Press).

22. Karen Barad (2010), “Quantum Entanglements and Hauntological Relations of Inheritance: Dis/continuities, SpaceTime Enfoldings, and Justice-to-Come,” *Derrida Today* 3, no. 2: 240–68.

23. Guri Aarseth et al. (2019), “Acting by Persuasion— Values and Rhetoric in Medical Certificates of Work Incapacity: A Qualitative Document Analysis,” *Medical Humanities* 45: 60–6; Hande Güzel (2018), “Pain as Performance: Re-Virginisation in Turkey,” *Medical Humanities* 44, no. 2: 89–95.

24. Meredith Stone et al. (2018), “Care(less) Encounters: Early Maternal Distress and the Haunted Clinic,” *Subjectivity* 11, no. 2: 108–27.

25. Burke, “On (Not) Caring.”

26. George Szmukler (2008), “Treatment Pressures, Coercion and Compulsion in Mental Health Care,” *Journal of Mental Health* 17, no. 3: 229–31; Szmukler, “Compulsion and ‘Coercion’ in Mental Health Care.”

27. Ignaas Devisch (2011), “Progress in Medicine: Autonomy, Oughtonomy and Nudging,” *Journal of Evaluation in Clinical Practice* 17, no. 5: 857–61.

28. See also Bell, “Thwarting the Diseased Will.”

29. Beyond Blue, 2018, “Know When Anxiety Is Talking,” last modified 2019, https://www.beyondblue.org.au/the-facts/anxiety; Naomi Elkin-Jones, and David Grusovin (2018), “*How 'Mad' Are You?”* in *How ‘Mad’ Are You?* eds. N. Elkin-Jones and D. Grusovin (Sydney: SBS,); John Read et al. (2006), “Prejudice and Schizophrenia: A Review of the ‘Mental Illness Is an Illness Like Any Other’ Approach,” *Acta Psychiatrica Scandinavica* 114, no. 5: 303–18.

30. Bernice A. Pescosolido et al. (2010), “’A Disease Like Any Other’? A Decade of Change in Public Reactions to Schizophrenia, Depression, and Alcohol Dependence,” *The American Journal of Psychiatry* 167, no. 11: 1321–30.

31. Bell, “Thwarting the Diseased Will”; Talia Weiner and T. Weiner (2011), “The (Un)managed Self: Paradoxical Forms of Agency in Self-Management of Bipolar Disorder,” *Culture, Medicine and Psychiatry* 35 no. 4: 448–83.

32. Bell, “Thwarting the Diseased Will”; Felicity Callard et al. (2012), “Holding Blame at Bay.”

33. See Stephen Frosh (2016) on discussion of Judith Butler and Walter Benjamin (“Relationality in a Time of Surveillance: Narcissism, Melancholia, Paranoia,” *Subjectivity* 9, no. 1: 1–16; or the schizophrenogenic mother (Callard et al, “Holding Blame at Bay”).

34. See Stone et al., “Care(less) Encounters.”

35. Shildrick et al., “Messy Entanglements,” 53.

36. Kim Eaton et al. (2017), “Mothers’ Decisions to Disclose or Conceal Their Child’s Mental Health Disorder,” *Qualitative Health Research* 27, no. 11: 1628–39; Jeannette Pols (2003), *Care in Practice*; Reidun Norvoll et al. (2018), “Family Members’ Existential and Moral Dilemmas with Coercion in Mental Healthcare,” *Qualitative Health Research* 28, no. 6: 900–15.

37. Norvoll et al., 900–15

38. Callard et al, “Holding Blame at Bay.”

39. And elsewhere: Susan Pickard (2010), “The ‘Good Carer’: Moral Practices in Late Modernity” *Sociology* 44, no. 3 (2010): 471–87; Carol Thomas (1993), “De-Constructing Concepts of Care,” *Sociology* 27, no. 4: 649–69.

40. Mark Fisher (2014), *Ghosts of My Life: Writings on Depression, Hauntology and Lost Futures* (London: Zero Books).

41. Deborah Steinberg (2015), “The Bad Patient: Estranged Subjects of the Cancer Culture,” *Body &* Society 21, no. 3: 115–43.

42. Burke, “On (Not) Caring”; Val Goodwin et al. (2006), “Conflicting Agendas between Consumers and Carers: The Perspectives of Carers and Nurses,” *The International Journal of Mental Health Nursing* 15 no. 2: 135–43; Janet Heaton (1999), “The Gaze and Visibility of the Carer: A Foucauldian Analysis of the Discourse of Informal Care,” *Sociology of Health & Illness* 21, no. 6: 759–77.

43. Fitzgerald and Callard, “Entangling the Medical Humanities.”

44. For a critique of expectations on informal carers, see also Liz Lloyd (2006), “Call Us Carers: Limitations and Risks in Campaigning for Recognition and Exclusivity,” *Critical Social Policy* 26, no. 4: 945–60; Mary Larkin and Alisoun Milne (2014), “Carers and Empowerment in the UK: A Critical Reflection,” *Social Policy and Society* 13, no. 1: 25–38; Lesley Murray and Marian Barnes (2010), “Have Families Been Rethought? Ethic of Care, Family and ‘Whole Family’ Approaches,” *Social Policy and Society* 9, no. 4: 533–44.

45. Vic.gov (2018), “Working Together with Families and Carers,” last modified 2018, https://www2.health.vic.gov.au/about/publications/policiesandguidelines/chief-psychiatrist-families-and-carer

46. Shildrick and Steinberg, “Estranged Bodies.”

47. Stone et al, “Care(less) Encounters.”

48. See Ignaas Devisch (2011), “Progress in Medicine”; Szmukler, “Treatment Pressures, Coercion and Compulsion.”

49. Ana Dragolic and Alex Broom, 2018, *Bodies and Suffering: Emotions and Relations of Care Bodies and Suffering* (London: Routledge): Stone et al, “Care(less) Encounters.”

50. Cecilia Benoit and Helga Hallgrimsdóttir (2011), *Valuing Care Work: Comparative Perspectives* (Toronto: University of Toronto Press).

51. Murray, “Afterword,” 627.

52. See also Steinberg, “The Bad Patient.”

53. See Renata Kokanović and Meredith Stone (2018), “Listening to What Cannot Be Said: Broken Narratives and the Lived Body,” *Arts and Humanities in Higher Education* 17, no. 1: 20–31.

54. See for example, Gooding, “Supported Decision-Making.”

55. Mol, *The Logic of Care*.

56. Mol, *The Logic of Care*; Pols, “Enforcing Patient Rights or Improving Care?”

57. See Stephen Allison et al. (2018), “Victoria’s Low Availability of Public Psychiatric Beds and the Impact on Patients, Carers and Staff,” *Australian and New Zealand Journal of Psychiatry* 52, no. 1: 91–2.

58. Antoine Hennion and Pierre A Vidal-Naquet, 2017, “Might Constraint Be Compatible with Care?”; Mol, *The Logic of Care*; Mol et al, *Care in Practice*.

59. Hennion and Vidal-Naquet, “Might Constraint Be Compatible with Care?”

60. Dragolic and Broom, *Bodies and Suffering*.

61. Vic.gov, “Mental Health Act 2014.”

62. Benjamin Noys (2010), *The Persistence of the Negative: A Critique of Contemporary Continental Theory* (London: Edinburgh University Press).

63. Mol, *T* *he Logic of Care*; Mol et al, *Care in Practice*.

64. Pols, “Enforcing Patient Rights or Improving Care?”

65. Hennion and Vidal-Naquet, “Might Constraint Be Compatible with Care?”

66. See, for example, Shildrick et al., “Messy Entanglements.”

67. Anne Whitehead and Angela Woods (2016), “Introduction,” in *The Edinburgh Companion to the Critical Medical Humanities*, eds. A. Whitehead, A. Woods, S. Atkinson, J. Macnaughton and S. Richards (Edinburgh: Edinburgh University Press), 1–32.
